# Assessing the seasonal prevalence and risk factors for nuchal crest adiposity in domestic horses and ponies using the Cresty Neck Score

**DOI:** 10.1186/s12917-015-0327-7

**Published:** 2015-01-31

**Authors:** Sarah L Giles, Christine J Nicol, Sean A Rands, Patricia A Harris

**Affiliations:** School of Veterinary Science, University of Bristol, Langford, Bristol, BS40 5DU UK; School of Biological Sciences, University of Bristol, Bristol Life Science Building, 24 Tyndall Avenue, Bristol, BS8 1TQ UK; WALTHAM Centre for Pet Nutrition, Equine Studies Group, Freeby Lane, Waltham-on-the-Wolds, Leicestershire, LE14 4RT Melton Mowbray UK

**Keywords:** Horse, Pony, Equine, Cresty neck, Cresty neck score, Obesity, Season, Body condition, Prevalence, Risk factors

## Abstract

**Background:**

Nuchal crest adiposity in horses and ponies has been associated with an enhanced risk of metabolic health problems. However, there is no current information on the prevalence of, and risk factors specific to, nuchal crest adiposity in horses and ponies. In addition, the cresty neck score has not previously been utilised across different seasons within a UK leisure population, it is not know whether nuchal crest adiposity shows the same seasonal trends as general obesity.

**Results:**

A Cresty Neck Score (CNS, 0–5) was given to 96 horses with access to pasture (>6 h per day) at the end of winter and at the end of summer in order to obtain two prevalence estimates. Risk factors were assessed using the single outcome cresty neck/no cresty neck in either season (binary), from owner questionnaires and analysed using a mixed effects logistic regression model (outcome variable CNS <3 or CNS ≥3/5). Agreement between winter and summer scores was assessed using weighted Kappa methods.

Winter CNS values were significantly higher than summer CNS values (p = 0.002) indicating a systematic bias. The prevalence of a CNS ≥ 3/5 was 45.83% at the end of winter, falling to 33.33% at the end of summer and was higher in ponies (<14.2 hh) than horses (≥14.2 hh) in both seasons. This may reflect a real winter increase in regional fat deposition, or an increased difficulty in obtaining an accurate estimate of regional adiposity in winter months. Breed was the strongest risk factor for CNS ≥3/5 in both seasons, with native UK breeds appearing to be most at risk (p < 0.001). In a separate, small validation study, the CNS showed good inter-observer reliability.

**Conclusions:**

The prevalence of a CNS ≥3/5 was higher at the end of winter than at the end of summer, which was the opposite pattern seasonal variation to that observed for general obesity. Further studies are required to investigate the potential influence of time of year upon CNS interpretation and studies utilising the CNS should consider potential seasonal variability in nuchal crest adiposity.

**Electronic supplementary material:**

The online version of this article (doi:10.1186/s12917-015-0327-7) contains supplementary material, which is available to authorized users.

## Background

It has become recognised in the human obesity literature that some patterns of regional fat accumulation have particularly damaging health consequences. Abdominal fat in humans has been linked to changes in circulating blood glucose levels, and fat accumulation in this area is a risk factor for insulin resistance, diabetes and other metabolic complications [1-4].

Increased fat deposits along the crest of the neck in horses and ponies (nuchal crest adiposity) has similarly been associated with an altered metabolic state [5,6] and an increased risk of certain metabolic disorders such insulin resistance [7-9]. A scoring system which targets this region specific adiposity has been developed by Carter and colleagues [5], named the ‘Cresty Neck Score’ (CNS), and in several studies a CNS score of ≥ 3/5 has been associated with an increased risk of laminitis [7,10,11]. It is not currently known whether this association with laminitis is due to the presence of additional fat in this nuchal crest region (potentially resulting in a more inflammatory profile [12,13]) or whether nuchal crest adiposity is a proxy for e.g. increased abdominal fat (potentially leading to abnormal insulin dynamics [12]) which is not detected via external Body Condition Scoring (BCS) systems. It has been suggested that certain breeds may more at risk of nuchal crest adiposity and associated metabolic disorders [6,14], particularly pony breeds [15], but evidence is currently limited.

The Cresty Neck Score [5] has become the most commonly used method of distinguishing nuchal crest adiposity [16-19]. Whilst this method has not been formally validated using post-mortem methods, there is a lack of gold standard measure for this type of specific, regional adiposity. Previously authors have utilised a neck circumference [8,12,20], but due to a lack of reference as to what a cresty neck or non-cresty neck circumference should be for any particular breed or size of horse or pony, this measure is only really useful as a ratio to neck length, or basis for assessing change, and thus has obvious limitations.

Previous studies have explored the prevalence of and risk factors for obesity in horses and ponies [21-26]. The prevalence of general obesity has been shown to vary between winter and summer in UK horses and ponies, with lower values at the end of winter [25]. It is not known whether the prevalence of CNS ≥3/5 differs from general obesity, or whether there is similar seasonal variation. Our hypothesis is that there would thus be similar seasonal changes in neck crest adiposity, with higher values during summer months. Neck crest adiposity may be a site of long-term fat storage, with previous studies reporting that fat reserves in this region (along with the rump) appear not to vary greatly with short term changes in energy intake at different times of the year [26]. It is therefore possible that the pattern of seasonal change observed in neck crest adiposity may be different from that seen with more general measures of obesity.

The overall aims of this study were: 1) To assess the prevalence of CNS ≥3/5, both at the end of winter and at the end of summer in a domestic population with daily access to pasture; 2) To examine the risk factors associated with CNS ≥3/5; and 3) To use seasonal CNS measurements from the same individual horses and ponies, and the same human observer, to assess the between-season agreement of the CNS.

## Methods

### Animal and materials

The study population was outdoor, herd-living leisure horses in North Somerset, UK. Making the assumption that seasonal body condition trends are clearest in outdoor living animals, inclusion criteria specified that horses and ponies had to live outdoors on green pasture for at least six hours per day. The target population was leisure horses and ponies in the UK, with daily access to pasture, the study population was thus likely to be fairly representative of this. The sampling frame consisted of horses and ponies whose owners attended a riding club membership renewals evening in January 2011. A cluster sampling strategy was used, where herds were randomly selected from the sampling frame using random number tables and all individuals were measured in that herd. 127 individual animals were initially recruited and measured between 5^th^ February – 24^th^ March 2011. Of these, 96 individuals remained with the same owners and were therefore were available for follow up. Second measurements were taken between 20^th^ July – 1^st^ September 2011 in these 96 individuals, which made up our study population.

Data on risk factors was obtained via two owner questionnaires which were completed at the same time as measurements were taken (for questionnaires see Additional file 1 in [25]). Owners completed risk factors questionnaires alone and in their own time to minimise observer bias. Questionnaires covered individual animal information such as breed and age, as well as management risk factors such as grazing and turnout routine, rug wearing, dental history, as well as a detailed breakdown of supplementary feed and exercise. Grass quality could not be measured due to horses moving pasture several times throughout the duration of the study and management routines such as strip grazing meant the quality of the grass forage varied daily. Height was measured at the withers using a measuring stick with spirit level. Horses and ponies were defined according to their height, where horses were >148 cm (equivalent to 14.2 hh) and ponies were ≤148 cm. Baseline descriptive statistics and study population demographics are presented in our related paper [25].

**Figure 1:**
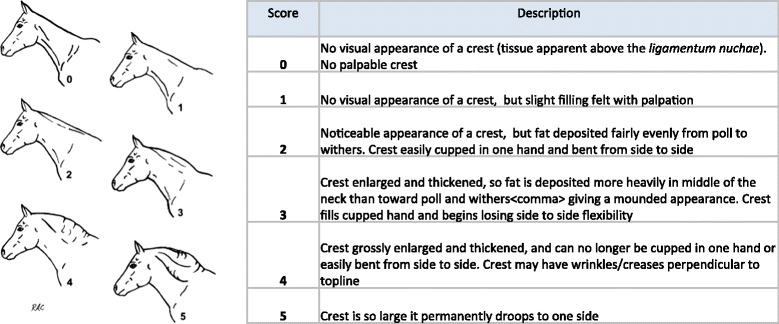
**The cresty neck score, descriptions and illustrations.** Reprinted from Carter RA, Geor RJ, Burton Staniar W, Cubuitt TA, Harris PA: Apparent adiposity assessed by standardised scoring systems and morphometric measurements in horses and ponies. *Vet J* 2009, 179:204–210, Copyright 2009, with permission from Elsevier.

Nuchal adiposity was measured using the CNS [5] and scored by a single trained observer for both winter and summer measurements. This involved a visual assessment of the neck as well as a tactile assessment of the nuchal crest area. The Cresty Neck Score is shown in Figure 1. Animals were considered to be cases if they had a CNS of three or above in either season (a binary outcome variable), this is the cut-off value recommended by Carter et al. [5]. This binary variable was the outcome variable used for the risk factor analysis.

### Statistical analysis

For the risk factor analysis, data were initially checked and missing values dealt with using complete case analysis, where incomplete units were removed. Response rate to the questionnaires was 100%. Age was considered as both a continuous variable and as a binary variable (‘youngster’/‘not youngster’), where not young was defined as an adult animal over four years of age). Height was also considered as both continuous and binary, so as to compare horses (≥148 cm) and ponies (<148 cm). Breed was initially coded into seven categories: native ponies (except Shetland), Shetlands and miniature breeds, lightweight breeds (Arabians and Thoroughbreds), heavyweight draught horses, cob types, sports horse breeds, and ‘other’. Due to low sample size in both the heavyweight and miniature breed categories (just 4 individuals in each), Shetlands were added to the native ponies category and heavyweight breeds were added to ‘other’, giving five final breed categories.

Quantity of both supplementary feed and forage was coded into categories for analysis. The categories of supplementary feed represented: category 1, no supplementary feed at all; category 2, no energy-providing feed (concentrate) but vitamin and mineral supplements given; category 3, less than 1.5 kg day^−1^) of ‘concentrate’ feed; and category 4, at least 1.5 kg day^−1^ of concentrate feed. These categories were analysed separately for horses and ponies, to account for the size of the animal. Hay was split into 3 categories, none, small amount (less than 5 kg day^−1^), and large amount (at least 5 kg day^−1^).

Risk factor variables were initially analysed univariably using χ^2^ tests, analysis of variance (ANOVA) or linear regression (all two tailed). Variables with evidence of association with CNS ≥ 3/5 were taken forward into multivariable analysis (screening *p* value ≤ 0.07). A mixed effects logistic regression model was then forward-fitted using a positive stepwise approach, based on strength of univariable association. Explanatory variables were included as fixed effects and retained both on the basis of their *p* values and also on the strength of their contribution to the model fit, assessed using the likelihood ratio test. Herd group was included as a random effect, to account for the clustered study design. Collinearity was assessed using pairwise correlations and the likelihood ratio test, and the possibility of non-linear or quadratic associations was also considered and assessed using the likelihood ratio test for all continuous explanatory variables.

### Cresty neck score in summer and winter

The same animals were measured in both seasons (*n* = 96) by the same observer. The extent of agreement between these two sets of results was assessed, using a Bland-Altman plot on the paired winter and summer CNS scores. A Wilcoxon signed rank test was then used to assess whether or not the differences between winter and summer measurements were significant. A weighted Cohen’s kappa statistic indicating level of agreement was also generated (using a squared weighting system equivalent to 1 - {(*i*-*j*)/(*k*-1)}^2^, where *i* and *j* refer to the row and column index, and *k* is the maximum number of possible ratings). All statistical analyses were performed in *Stata* 11.2 (Statacorp, College Station, Texas).

### Inter-observer reliability of the cresty neck score

An additional 31 horses and ponies were recruited *post hoc* from the Royal Veterinary College, University of London, to obtain a measure of inter-observer reliability in summer CNS scores and to assess this as a potential source of error. Made up of 18 ponies, native types and 13 horses, mostly Thoroughbred types. Inter-observer agreement has not previously been reported for this score. These 31 horses were deliberately a mixture of ages, breeds and body condition scores. Three trained observers, including SLG (observer 2) assigned a CNS to the individual animals and all data collection was completed on the same day, 29/07/2014. One observer only assessed 29 animals. All observers were blinded to the results of the other observers. The agreement between the observers was then assessed using Cohen’s kappa statistic using the weighting described above.

All experimental work was approved by the University of Bristol Ethical Review Group (university investigation number UB/10/049).

## Results

### Prevalence of CNS ≥ 3/5

The prevalence of CNS ≥3/5 within our study population (*n* = 96) varied between winter and summer measurements and there was strong evidence for a seasonal difference in nuchal crest adiposity (χ_1_^2^ = 16.45, p < 0.001). The prevalence was 45.83% (95% CI 36.76% - 54.90%) at the end of winter, falling to 33.33% (95% CI 26.76% - 39.90%) at the end of summer. Of these 96 individuals, 20 (20.83%) had a CNS ≥3/5 in the winter and not in the summer, whereas only 8 (8.33%) had a CNS ≥3/5 in the summer and not in the winter.

The 20 individuals which had a CNS ≥3/5 in winter but not in summer were evenly spread across both height and breed categories.

Table 1 shows the observed differences between horses and ponies, ponies had a higher prevalence of CNS ≥3/5 during both seasons. At the end of the winter months ponies had twice (2.00) the odds of CNS ≥3/5 compared to horses, but this was not significant. There was however strong evidence of a difference in the number of cases between horses and ponies at the end of the summer months (OR 2.83 *p* = 0.02).

**Table 1:** **A comparison of CNS ≥3/5 prevalence between horses and ponies during winter and summer**

**Winter**	**Total number of subjects (%)**	**Number of cases (%)**	**Odds ratio (ponies/horses)**	**95% CI**	**χ** ^**2**^	***p***
**Horses**	46 (47.92)	17 (38.64)	-	-		-
**Ponies**	50 (52.08)	27 (61.36)	2.00	0.87 – 4.61	2.77	0.09
**Total**	96 (100)	44 (45.83)				
**Summer**						
**Horses**	46 (47.92)	10 (31.25)	-	-		-
**Ponies**	50 (52.08)	22 (68.75)	2.83	112 – 7.14	5.29	0.02
**Total**	96 (100)	32 (33.33)				
**Odds ratio (winter/summer)**			0.71			

### Risk factors for CNS ≥3/5

Risk factors which showed an initial univariable association with nuchal crest adiposity are summarised in Table 2. Height was associated with CNS ≥3/5, both as a categorical and binary (horses/ponies) variable (*p* = 0.04 and *p* = 0.02 respectively), where ponies, and more specifically ponies between 114–134 cm appear to be at greatest risk. Horses or ponies with a history of laminitis were more likely to have CNS ≥3/5 (*p* = 0.04). Breed showed strong evidence of association (*p* < 0.001), native UK breeds had the highest odds of CNS ≥3/5. Although feeding supplementary hay during the winter months was relatively rare in our study population (6.38%), this increased the odds of CNS ≥3/5 (*p* = 0.06). Those individuals with a complete change in feeding regimen between seasons showed the highest odds of CNS ≥3/5 (*p* = 0.005) however this is likely to be an issue of cause and effect, through owners attempting (and failing) to implement preventative strategies in at risk individuals. Herd size was also associated with cresty neck (*p* = 0.01), as herd size increased there were fewer cresty neck cases (CNS ≥3/5).

**Table 2:** **Univariable risk factors associated with CNS ≥3/5**

**Exposure variables**							
		***Total number of horses and ponies (%)***	***Number of CNS ≥ 3/5 cases (%)***	***Odds (CNS <3/5/ CNS ≥ 3/5)***	***95% CI***	**χ** ^***2***^	***p***
***Height (cm, categorical)***	**≤113**	8 (8.33)	2 (6.25)	0.33	0.06 – 1.65		
	**114 – 134**	20 (20.83)	12 (37.5)	1.50	0.61 – 3.67		
	**135 – 146**	22 (22.92)	8 (25.0)	0.57	0.24 – 1.36		
	**147– 159**	22 (22.92)	5 (15.62)	0.29	0.11 – 0.80		
	**≥160**	24 (25.0)	5 (15.62)	0.26	0.09 – 0.70	9.91	0.04
***Breed (categorical)***	**Native ponies**	34 (35.42)	15 (46.88)	0.79	0.40 – 1.55		
	**Lightweight**	25 (26.04)	1 (3.12)	0.04	0.005 – 0.31		
	**Native cobs**	12 (12.50)	7 (21.88)	1.40	0.44 – 4.41		
	**Sports horse**	13 (13.54)	1 (3.12)	0.08	0.01 – 0.64		
	**Other**	12 (12.50)	8 (25.0)	2.0	0.60 – 6.64	–	<0.001*
***Seasonal change in feeding regimen***	**No change**	13 (13.98)	8 (25.0)	1.60	0.52 – 4.89		
	**Reduction in quantity only**	13 (13.98)	3 (9.38)	0.30	0.08 – 1.09		
	**Change Parts**	57 (61.29)	14 (43.75)	0.32	0.17 – 0.60		
	**Complete Change**	10 (10.75)	7 (21.88)	2.33	0.60 – 9.02	13.04	0.005
***Laminitis***	**No**	82 (89.13)	23 (79.31)	0.38	0.24 – 0.63		
	**Yes**	10 (10.87)	6 (20.69)	1.50	0.42 – 5.32	4.22	0.04
***Fed hay or other supplementary forage during winter months***	**No**	88 (93.62)	26 (86.67)	0.42	0.27 – 0.66		
	**Yes**	6 (6.38)	4 (13.33)	2.00	0.37 – 10.92	3.56	0.06
***Height binary***	**Pony (≤148 cm)**	50 (52.08)	22 (68.75)	0.78	0.44 – 1.37		
	**Horse (>148 cm)**	46 (47.92)	10 (31.25)	0.28	0.14 – 0.56	5.34	0.02
***Herd size***		96 (100)	-	-	-	−2.48	0.01**
***Total***		96 (100)	32 (100)				

*indicates probability estimated using Fisher’s exact test. **continuous variable assessed for a univariable association using logistic regression.

The final risk factor regression model for CNS ≥3/5 is presented in Table 3. The final model contained the variables, breed category and herd size. Native ponies had 19.72 times the odds of CNS ≥3/5 (*p* = 0.006), compared to lightweight breeds (as referent), and native cobs appear even more at risk with 31.48 times the odds (*p* = 0.004). As herd size increases, the odds of a CNS ≥3/5 are reduced by 18% per extra individual.

**Table 3:** **Final multivariable model for risk factors associated with CNS ≥3/5**

***Risk factor***	***Odds ratio***	***SE***	***95% CI***	***p***
***Breed***				
***Native ponies***	19.72	26.42	2.31 – 168.73	0.006
***Lightweight (baseline)***	1	-	-	-
***Native cobs***	31.48	37.61	3.03 – 327.30	0.004
***Sports horse***	1.75	2.58	0.09 – 31.34	0.70
***Other***	40.03	48.21	3.78 – 424.31	0.002
Herd size (number of individuals)*	0.87	0.05	0.77 – 0.98	0.02
***Constant***	0.10	0.12	0.01 – 0.92	0.04

*decrease CNS for every extra individual in the herd.

### Cresty neck score in summer and winter

**Figure 2:**
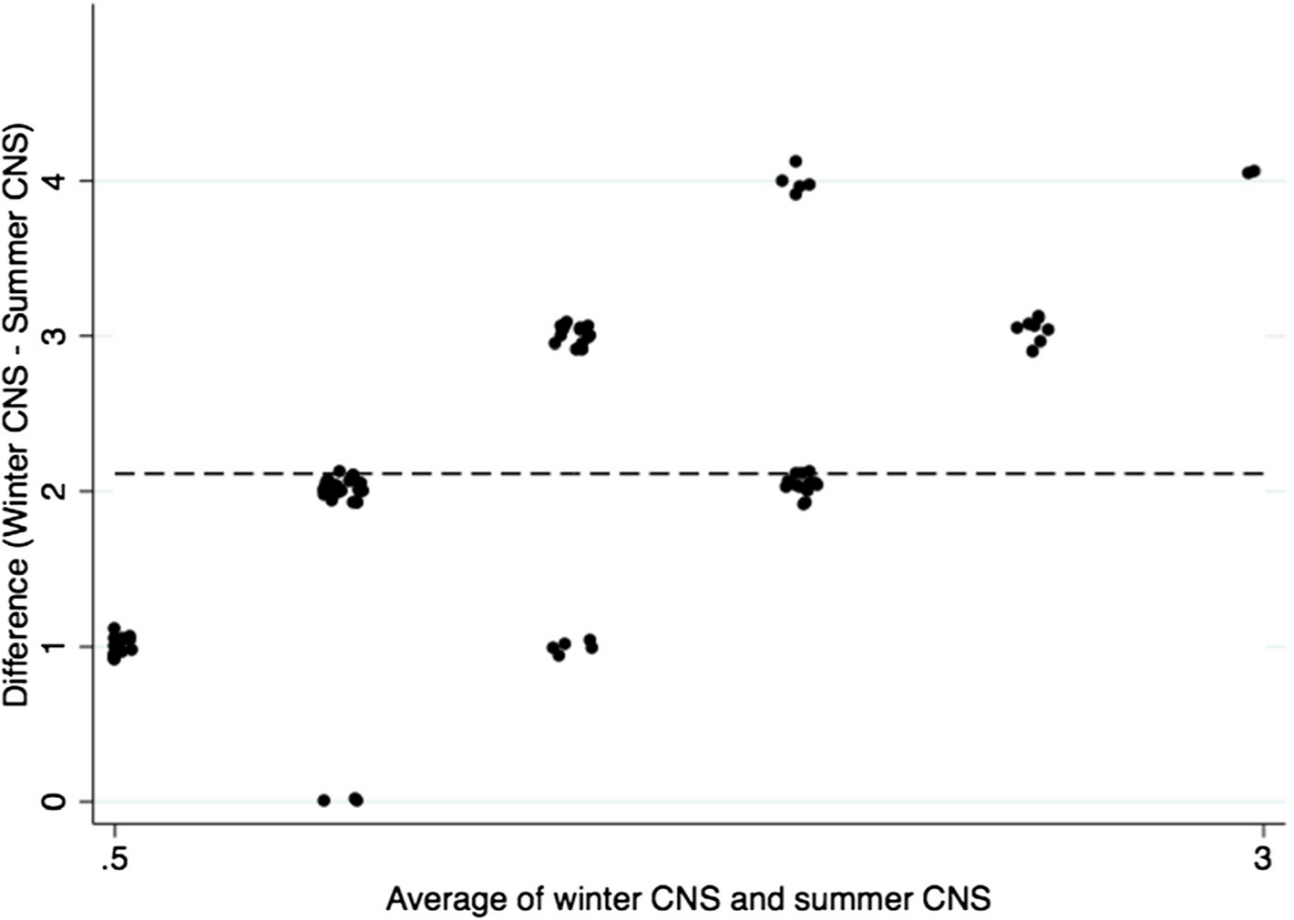
**Bland-Altman plot showing agreement between winter and summer CNS measures.**

Figure 2 shows the Bland-Altman plot for the extent of agreement between winter and summer CNS measures. The mean winter CNS score was 2.5 and the mean summer score was 1.9. A mean difference above of 0.32 (95% CI 0.12 – 0.52) indicates a consistent tendency for winter CNS measurements higher than summer CNS measurements, indicating a systematic bias.

There was strong evidence of a difference between winter CNS and summer CNS measures using a Wilcoxon signed rank test (*T* = 3.03, *N* =96, *p* = 0.002) confirming winter measurements were significantly higher. There was a moderate agreement between winter and summer CNS (κ_*w*_ = 0.51, *Z* = 5.25, *p* < 0.001).

### Inter-observer reliability of the cresty neck score

The inter-observer agreement of the three trained observers was good (Table 4), with moderate agreement between observers 1 and 2 and observers 1 and 3, and substantial agreement between observers 2 and 3. When the scores for horses and ponies were examined separately inter-observer reliability remained good, though agreement was lower for horses than ponies.

**Table 4:** Weighted Cohen’s kappa showing the inter-observer agreement results between the 3 trained observers assessing 31* horses and ponies

***Observers compared***	***Observed agreement (%)***	***Expected agreement (%)***	**κ**_***w***_	***SE***	***Z***	***p***
***Observer 1 and Observer 2***	95.86	89.57	0.60	0.16	3.69	<0.001
***Observer 2 and Observer 3***	97.16	90.28	0.71	0.17	4.09	<0.001
***Observer 1 and Observer 3***	94.61	88.30	0.54	0.16	3.26	0.001

*note one observer only assessed 29 horses and ponies.

## Discussion

The prevalence of CNS ≥3/5 was significantly higher at the end of the winter months, 45.83%, compared to 33.33% at the end of the summer. Nuchal neck adiposity therefore shows the opposite seasonal pattern to that previously demonstrated for general obesity in the same study population [25], where general obesity prevalence was found to be highest at the end of the summer months. This disproves our original hypothesis. There are two possible major reasons why neck crest adiposity differs to general adiposity in this way: this is either a real physiological effect, or a methodological anomaly with the Cresty Neck Score itself. First a potential methodological explanation will be considered, and then the biological results can be more clearly deliberated in light of this.

It is possible that the differences in the seasonal prevalences seen may be due to errors with our application of the CNS. There was a systematic variation between the winter and summer measures, where winter measures were significantly higher. Time of year appears to somehow affect the application and interpretation of this score. This was a non-blinded study, and so there is a potential that this could have biased the summer measurements, however even if observer bias did occur due to non-blinding, we would expect summer estimates to be an over rather than under-estimate, so this does not explain the results observed.

A lack of between-season agreement would not have been surprising in itself; it is the pattern of seasonal variation observed here which was unexpected. We argue that observer error is ultimately unlikely to fully explain the extent of differences in the prevalence of cresty neck observed between seasons in this study population. However we do suggest that errors with the between-season repeatability of the score may partly contributed as a minor influence. It may be that the CNS is less effective at distinguishing neck adiposity during the winter months when horses and ponies have a fluffy winter coat (although measuring neck circumference using a tape measure would encounter similar issues). A large study conducted during the winter months, containing a number of different breeds with varying winter coat lengths and BCS systems, would be required to investigate this. This was beyond the scope of this particular study. As the CNS is a relatively new measure, and the seasonal variation in nuchal crest adiposity has not previously been investigated. These results may therefore have important implications for the future application of the CNS, particularly with regards to considering potential seasonal variability. Based on the between-season variability observed here , we would recommend that alongside an investigation of the influence of breed, the repeatability of the Cresty Neck Score be tested using test-retest methods at several key seasonal time points, to see if it is a suitable measure for such investigations. It may be that this measure is not appropriate to compare adiposity in outdoor living animals measured at different times of year.

As far as we are aware this is first study to have investigated the inter-observer reliability of the CNS score. Notably the agreement between observers, at a single moment in time, was greater than agreement between the results of the same observer at the two different seasonal time points. It was reassuring to discover that all observers had good agreement, indicating that the score has good inter-observer repeatability. However the systematic variation in scores observed may have other implications. Whilst discrepancies by just one scoring level would not contribute greatly to a perceived ‘lack of agreement’ using weighted kappa methods, they may have more serious clinical consequences. An observer that scores 2 instead of 3, or *vice versa*, could inadvertently alter the results of a clinical study dramatically if this is the cut-off criterion being used to distinguish a ‘cresty neck’ case from a non-‘cresty neck’ case. These two scores are the most commonly used by observers, occupying the middle of the scorable range. There is therefore still the possibility, regardless of the generally good agreement scores, that the final results were influenced by the cut-off used. Further investigations are certainly required to fully understand the possible implications of this.

Despite this systematic difference between winter and summer measures, our results suggest that the reliability of the CNS is generally good. It therefore is just as likely that there is a biological explanation for the pattern of seasonal variation observed in nuchal crest adiposity. It is possible that nuchal cresty adiposity could have a functional physiological role as an indicator of individuals coming out of winter in good breeding condition, but this is just speculation. Previous authors have suggested that tissue within the nuchal region in horses and ponies is akin to visceral abdominal fat in humans [10,27]. If nuchal fat is indeed similar to abdominal fat in humans, then the type of fat stored in the neck crest may be structurally different, and have different functional properties to that of fat stored elsewhere. In humans, visceral abdominal fat is more strongly associated with metabolic abnormalities such as type-2 diabetes mellitus, insulin resistance and hyperinsulinemia [1-4]. It has been suggested that adipose tissue in this region represents ‘dysfunctional’ adipose tissue in individuals unable to store excess energy effectively [28]. In humans this defect in energy partitioning leads to an altered metabolic profile and inflammatory state, and the same could be true in horses and ponies with excess nuchal crest adiposity.

Evidence regarding these physiological mechanisms in horses is still mixed. Burns and colleagues [12] found no evidence of a difference in gene expression for proinflammatory cytokines in the nuchal (neck crest) region between insulin resistant and insulin sensitive animals but did find higher levels of some interleukins (IL-1β and IL-6) in the nuchal crest region suggesting it was a more active proinflammatory cytokine depot. Bruynsteen and colleagues [13] found evidence of higher leptin mRNA expression in adipose tissue from the nuchal region compared to mesenteric adipose tissue, but in contrast to Burns et al. [12], the highest levels of IL-1β were found in abdominal adipose tissue, suggesting that this region, similarly to humans, may in fact be more important than the nuchal crest adipose tissue in determining circulating levels of cytokines. Potentially nuchal crest adiposity could simply be a proxy measure which is associated with increased internal abdominal adiposity not detectable by visual body condition scoring methods. Note that both of these studies rely on gene expression and thus results may be influenced by the reference genes chosen, studies relating directly to protein expression and circulating proinflammatory cytokines are clearly required to further investigate these mechanisms.

In horses and ponies, insulin resistance has been strongly associated with neck crest adiposity and generalised obesity [7,8,11]. Insulin sensitivity, like body condition, has also been suggested to vary seasonally [7,14,29,30] especially in hardy native pony breeds, in which the prevalence of nuchal crest adiposity was highest in the current study. Studies have shown a higher insulin sensitivity during the autumn and winter and a lower insulin sensitivity during the spring and summer [7,30]. Interestingly the results presented here show the same seasonal physiological pattern in nuchal crest adiposity. As we did not measure insulin sensitivity in these animals, any discussion of insulin responsiveness here is purely speculative.

The neck crest area of fat storage takes longer to develop and deplete than other fat storage sites in horses and ponies [6,26]. Changes in nuchal crest adiposity may therefore not fit within seasonal patterns of food availability and may instead reflect longer-term management trends. This could offer another explanation as to why the seasonal variation in nuchal crest adiposity is different to that of general obesity.

We examined whether the higher winter prevalence of CNS ≥3/5 could be explained by exercise. On average ponies carried out less exercise than horses and fewer ponies carried out any form of structured exercise (see [25]). It is possible this exacerbated the higher prevalence of CNS ≥3/5 in ponies over horses. On average, horses and ponies that were ridden received around 1.5 hours more exercise per week during the summer months than the winter months (see [25]). These seasonal changes in structured exercise may act to reduce nuchal crest adiposity during the summer months through a combination of higher amounts of exercise alongside increased metabolic rate and improved insulin sensitivity. Although exercise did not appear to explain variation within nuchal crest adiposity in our study population, but a high percentage (over 60%) did not carry out any regular structured exercise.

The prevalence of CNS ≥3/5 in ponies at the end of winter was 61%, representing a real potential welfare issue if a CNS ≥3/5 is shown to be truly associated with an increase risk of serious health consequences. Risk factor analysis revealed that native cobs as well as native ponies appear to have a strong breed predisposition to nuchal crest adiposity. This is similar to the breed association seen with general obesity [25]. This demonstrates that breed needs to be considered when CNS measurements are taken. Previous authors have also suggested a breed predisposition to an enlarged nuchal crest [6,15], and our results support this. Native UK breeds naturally live in nutritionally sparse winter environments, such as on mountains or moorland [31], and nuchal crest adiposity may therefore be an adaptive mechanism of fat storage for survival during periods of nutritional scarcity. These results support this, the described adaptive strategy may be less clear in domestically selected lines, with sports horse and lightweight breeds appearing to be at a lessened risk . It must be noted that lightweight breeds within this particular study population of UK leisure horses, refers mostly to Arabian and Thoroughbred types and there were few American stock horse or Spanish breeds, which previous authors have suggested may also be at higher risk of nuchal crest adiposity [6].

It must be considered however, that whilst the genetics of these native breeds may be similar to their wild or feral counterparts, and thus possess similar seasonal fluctuations in body condition, the management of domestic native breeds is often very different to their ‘natural’ environment, with food often plentiful year round. The seasonal pattern of nuchal crest variation observed here, could therefore just be a consequence of the unnaturally nutrient rich captive environment in which domestic native breeds are kept. It would certainly be of interest to investigate seasonal variation in nuchal crest adiposity in feral herds. The results of this study also focus purely on a study population with daily access to pasture, it is not know how applicable these results would be to horses and ponies kept in a predominantly stabled environment, which plausibly do not have the same seasonal variation in food supply and body condition.

The effect of herd size upon body condition in horses has not previously been reported. Our results suggest that as the number of horses in the herd increase, there is an apparent decrease in neck crest adiposity. With an increased group size there is likely to be greater competition between individuals for forage resources, individuals are likely to be interrupted from grazing more often due to socially mediated interference [32] and spontaneous activity levels may also be higher in larger groups. It was noted that generally large groups did have a larger pasture area, as would be expected, but this was not measured explicitly. An alternate management related explanation could be that laminitis prone horses and ponies, which are also likely to be those with the largest neck circumference, may be removed from the main herd or managed separately in smaller groups on smaller pasture areas.

## Conclusions

Our study demonstrates that winter and summer cresty neck scores vary within the same population of animals. Several possible explanations for this have been outlined. These differences could represent a physiological phenomena, where neck crest fat is larger at the end of winter and depletes during the summer possibly due to a higher metabolic rate, higher activity and possibly a change in insulin sensitivity. Alternatively these results could represent an anomaly with the CNS itself. Further studies are required to validate the score for clinical research use, especially in outdoor living horses and ponies. Using the CNS system alongside the Henneke et al. [33] BCS system may enable potentially different types of obesity with different risks for health to be monitored more accurately.

## Additional file

Additional file 1:
**Risk factor questionnaires.**

## Abbreviations

CNS: Cresty neck score
BCS: Body condition score
