# Supplementary material for: Assessing the seasonal prevalence and risk factors for nuchal crest adiposity in domestic horses and ponies using the Cresty Neck Score
Source: BMC Vet Res. 2015 Jan 31;11:13. doi: 10.1186/s12917-015-0327-7 (PMC4347557; doi:10.1186/s12917-015-0327-7)
Supplement: Additional file 1: — Risk factor questionnaires. [file 12917_2015_327_MOESM1_ESM.docx]

| **ELSEVIER LICENSE TERMS AND CONDITIONS** | |  |  |
| --- | --- | --- | --- |
| Aug 28, 2014 | |  |  |
| This is a License Agreement between Sarah Giles ("You") and Elsevier ("Elsevier") provided by Copyright Clearance Center ("CCC"). The license consists of your order details, the terms and conditions provided by Elsevier, and the payment terms and conditions. | |  |  |
| **All payments must be made in full to CCC. For payment instructions, please see information listed at the bottom of this form.** | |  |  |
| Supplier | Elsevier Limited The Boulevard,Langford Lane Kidlington,Oxford,OX5 1GB,UK |  |  |
| Registered Company Number | 1982084 |  |  |
| Customer name | Sarah Giles |  |  |
| Customer address | Langford House |  |  |
|  | Bristol, BS40 5DU |  |  |
| License number | 3457640454910 |  |  |
| License date | Aug 28, 2014 |  |  |
| Licensed content publisher | Elsevier |  |  |
| Licensed content publication | The Veterinary Journal |  |  |
| Licensed content title | Apparent adiposity assessed by standardised scoring systems and morphometric measurements in horses and ponies |  |  |
| Licensed content author | Rebecca A. Carter,Raymond J. Geor,W. Burton Staniar,Tania A. Cubitt,Pat A. Harris |  |  |
| Licensed content date | February 2009 |  |  |
| Licensed content volume number | 179 |  |  |
| Licensed content issue number | 2 |  |  |
| Number of pages | 7 |  |  |
| Start Page | 204 | | |
| End Page | 210 | | |
| Type of Use | reuse in a journal/magazine | |  |
| Requestor type | author of new work | |  |
| Intended publisher of new work | Springer Science+Business Media | |  |
| Portion | figures/tables/illustrations | |  |
| Number of figures/tables/illustrations | 2 | |  |
| Format | both print and electronic | |  |
| Are you the author of this Elsevier article? | Yes | |  |
| Will you be translating? | No | |  |
| Title of the article | Assessing the seasonal prevalence and risk factors for nuchal crest adiposity in domestic horses and ponies using the Cresty Neck Score | |  |
| Publication new article is in | BMC Veterinary Research | |  |
| Publisher of the new article | Springer Science+Business Media | |  |
| Author of new article | Sarah L. Giles, Christine J. Nicol, Sean A. Rands and Patricia A. Harris | |  |
| Expected publication date | Jan 2015 | |  |
| Estimated size of new article (number of pages) | 13 | |  |
| Elsevier VAT number | GB 494 6272 12 | | |
| Permissions price | 0.00 GBP | |  |
| VAT/Local Sales Tax | 0.00 GBP / 0.00 GBP | | |
| Total | 0.00 GBP |  |  |
| Terms and Conditions | |  |  |
| **INTRODUCTION**  1. The publisher for this copyrighted material is Elsevier.  By clicking "accept" in connection with completing this licensing transaction, you agree that the following terms and conditions apply to this transaction (along with the Billing and Payment terms and conditions established by Copyright Clearance Center, Inc. ("CCC"), at the time that you opened your Rightslink account and that are available at any time at[http://myaccount.copyright.com](http://myaccount.copyright.com/)).  **GENERAL TERMS**  2. Elsevier hereby grants you permission to reproduce the aforementioned material subject to the terms and conditions indicated.  3. Acknowledgement: If any part of the material to be used (for example, figures) has appeared in our publication with credit or acknowledgement to another source, permission must also be sought from that source.  If such permission is not obtained then that material may not be included in your publication/copies. Suitable acknowledgement to the source must be made, either as a footnote or in a reference list at the end of your publication, as follows:  “Reprinted from Publication title, Vol /edition number, Author(s), Title of article / title of chapter, Pages No., Copyright (Year), with permission from Elsevier [OR APPLICABLE SOCIETY COPYRIGHT OWNER].” Also Lancet special credit - “Reprinted from The Lancet, Vol. number, Author(s), Title of article, Pages No., Copyright (Year), with permission from Elsevier.”  4. Reproduction of this material is confined to the purpose and/or media for which permission is hereby given.  5. Altering/Modifying Material: Not Permitted. However figures and illustrations may be altered/adapted minimally to serve your work. Any other abbreviations, additions, deletions and/or any other alterations shall be made only with prior written authorization of Elsevier Ltd. (Please contact Elsevier at permissions@elsevier.com)  6. If the permission fee for the requested use of our material is waived in this instance, please be advised that your future requests for Elsevier materials may attract a fee.  7. Reservation of Rights: Publisher reserves all rights not specifically granted in the combination of (i) the license details provided by you and accepted in the course of this licensing transaction, (ii) these terms and conditions and (iii) CCC's Billing and Payment terms and conditions.  8. License Contingent Upon Payment: While you may exercise the rights licensed immediately upon issuance of the license at the end of the licensing process for the transaction, provided that you have disclosed complete and accurate details of your proposed use, no license is finally effective unless and until full payment is received from you (either by publisher or by CCC) as provided in CCC's Billing and Payment terms and conditions.  If full payment is not received on a timely basis, then any license preliminarily granted shall be deemed automatically revoked and shall be void as if never granted.  Further, in the event that you breach any of these terms and conditions or any of CCC's Billing and Payment terms and conditions, the license is automatically revoked and shall be void as if never granted.  Use of materials as described in a revoked license, as well as any use of the materials beyond the scope of an unrevoked license, may constitute copyright infringement and publisher reserves the right to take any and all action to protect its copyright in the materials.  9. Warranties: Publisher makes no representations or warranties with respect to the licensed material.  10. Indemnity: You hereby indemnify and agree to hold harmless publisher and CCC, and their respective officers, directors, employees and agents, from and against any and all claims arising out of your use of the licensed material other than as specifically authorized pursuant to this license.  11. No Transfer of License: This license is personal to you and may not be sublicensed, assigned, or transferred by you to any other person without publisher's written permission.  12. No Amendment Except in Writing: This license may not be amended except in a writing signed by both parties (or, in the case of publisher, by CCC on publisher's behalf).  13. Objection to Contrary Terms: Publisher hereby objects to any terms contained in any purchase order, acknowledgment, check endorsement or other writing prepared by you, which terms are inconsistent with these terms and conditions or CCC's Billing and Payment terms and conditions.  These terms and conditions, together with CCC's Billing and Payment terms and conditions (which are incorporated herein), comprise the entire agreement between you and publisher (and CCC) concerning this licensing transaction.  In the event of any conflict between your obligations established by these terms and conditions and those established by CCC's Billing and Payment terms and conditions, these terms and conditions shall control.  14. Revocation: Elsevier or Copyright Clearance Center may deny the permissions described in this License at their sole discretion, for any reason or no reason, with a full refund payable to you.  Notice of such denial will be made using the contact information provided by you.  Failure to receive such notice will not alter or invalidate the denial.  In no event will Elsevier or Copyright Clearance Center be responsible or liable for any costs, expenses or damage incurred by you as a result of a denial of your permission request, other than a refund of the amount(s) paid by you to Elsevier and/or Copyright Clearance Center for denied permissions.  **LIMITED LICENSE**  The following terms and conditions apply only to specific license types:  15.**Translation**: This permission is granted for non-exclusive world **English** rights only unless your license was granted for translation rights. If you licensed translation rights you may only translate this content into the languages you requested. A professional translator must perform all translations and reproduce the content word for word preserving the integrity of the article. If this license is to re-use 1 or 2 figures then permission is granted for non-exclusive world rights in all languages.  16.**Posting licensed content on any Website**: The following terms and conditions apply as follows: Licensing material from an Elsevier journal: All content posted to the web site must maintain the copyright information line on the bottom of each image; A hyper-text must be included to the Homepage of the journal from which you are licensing at<http://www.sciencedirect.com/science/journal/xxxxx> or the Elsevier homepage for books at[http://www.elsevier.com](http://www.elsevier.com/); Central Storage: This license does not include permission for a scanned version of the material to be stored in a central repository such as that provided by Heron/XanEdu.  Licensing material from an Elsevier book: A hyper-text link must be included to the Elsevier homepage at [http://www.elsevier.com](http://www.elsevier.com/) . All content posted to the web site must maintain the copyright information line on the bottom of each image.  **Posting licensed content on Electronic reserve**:  In addition to the above the following clauses are applicable: The web site must be password-protected and made available only to bona fide students registered on a relevant course. This permission is granted for 1 year only. You may obtain a new license for future website posting.  **For journal authors:** the following clauses are applicable in addition to the above: Permission granted is limited to the author accepted manuscript version* of your paper.  ***Accepted Author Manuscript** **(AAM)** **Definition:** An accepted author manuscript (AAM) is the author’s version of the manuscript of an article that has been accepted for publication and which may include any author-incorporated changes suggested through the processes of submission processing, peer review, and editor-author communications. AAMs do not include other publisher value-added contributions such as copy-editing, formatting, technical enhancements and (if relevant) pagination.  You are not allowed to download and post the published journal article (whether PDF or HTML, proof or final version), nor may you scan the printed edition to create an electronic version. A hyper-text must be included to the Homepage of the journal from which you are licensing at <http://www.sciencedirect.com/science/journal/xxxxx>. As part of our normal production process, you will receive an e-mail notice when your article appears on Elsevier’s online service ScienceDirect (www.sciencedirect.com). That e-mail will include the article’s Digital Object Identifier (DOI). This number provides the electronic link to the published article and should be included in the posting of your personal version. We ask that you wait until you receive this e-mail and have the DOI to do any posting.  **Posting to a repository:** Authors may post their AAM immediately to their employer’s institutional repository for internal use only and may make their manuscript publically available after the journal-specific embargo period has ended.  Please also refer to [Elsevier's Article Posting Policy](http://www.elsevier.com/about/open-access/open-access-policies/article-posting-policy) for further information.  18.**For book authors**the following clauses are applicable in addition to the above:   Authors are permitted to place a brief summary of their work online only.. You are not allowed to download and post the published electronic version of your chapter, nor may you scan the printed edition to create an electronic version. **Posting to a repository:**Authors are permitted to post a summary of their chapter only in their institution’s repository.  20.**Thesis/Dissertation**: If your license is for use in a thesis/dissertation your thesis may be submitted to your institution in either print or electronic form. Should your thesis be published commercially, please reapply for permission. These requirements include permission for the Library and Archives of Canada to supply single copies, on demand, of the complete thesis and include permission for UMI to supply single copies, on demand, of the complete thesis. Should your thesis be published commercially, please reapply for permission.    **Elsevier Open Access Terms and Conditions**  Elsevier publishes Open Access articles in both its Open Access journals and via its Open Access articles option in subscription journals.  Authors publishing in an Open Access journal or who choose to make their article Open Access in an Elsevier subscription journal select one of the following Creative Commons user licenses, which define how a reader may reuse their work: Creative Commons Attribution License (CC BY), Creative Commons Attribution – Non Commercial -ShareAlike (CC BY NC SA) and Creative Commons Attribution – Non Commercial – No Derivatives (CC BY NC ND)  **Terms & Conditions applicable to all Elsevier Open Access articles:**  Any reuse of the article must not represent the author as endorsing the adaptation of the article nor should the article be modified in such a way as to damage the author’s honour or reputation.  The author(s) must be appropriately credited.  If any part of the material to be used (for example, figures) has appeared in our publication with credit or acknowledgement to another source it is the responsibility of the user to ensure their reuse complies with the terms and conditions determined by the rights holder.  **Additional Terms & Conditions applicable to each Creative Commons user license:**  **CC BY:** You may distribute and copy the article, create extracts, abstracts, and other revised versions, adaptations or derivative works of or from an article (such as a translation), to include in a collective work (such as an anthology), to text or data mine the article, including for commercial purposes without permission from Elsevier  **CC BY NC SA:** For non-commercial purposes you may distribute and copy the article, create extracts, abstracts and other revised versions, adaptations or derivative works of or from an article (such as a translation), to include in a collective work (such as an anthology), to text and data mine the article and license new adaptations or creations under identical terms without permission from Elsevier  **CC BY NC ND:** For non-commercial purposes you may distribute and copy the article and include it in a collective work (such as an anthology), provided you do not alter or modify the article, without permission from Elsevier  Any commercial reuse of Open Access articles published with a CC BY NC SA or CC BY NC ND license requires permission from Elsevier and will be subject to a fee.  Commercial reuse includes:  •         Promotional purposes (advertising or marketing)  •         Commercial exploitation ( e.g. a product for sale or loan)  •         Systematic distribution (for a fee or free of charge)  Please refer to [Elsevier's Open Access Policy](http://www.elsevier.com/about/open-access/open-access-policies/oa-license-policy) for further information.    21.**Other Conditions**:    v1.6 | |  |  |
| **You will be invoiced within 48 hours of this transaction date. You may pay your invoice by credit card upon receipt of the invoice for this transaction. Please follow instructions provided at that time.  To pay for this transaction now; please remit a copy of this document along with your payment. Payment should be in the form of a check or money order referencing your account number and this invoice number RLNK501388880. Make payments to "COPYRIGHT CLEARANCE CENTER" and send to:   Copyright Clearance Center Dept 001 P.O. Box 843006 Boston, MA 02284-3006 Please disregard electronic and mailed copies if you remit payment in advance. Questions?**[**customercare@copyright.com**](mailto:customercare@copyright.com)**or +1-855-239-3415 (toll free in the US) or +1-978-646-2777.** | |  |  |
| **Gratis licenses (referencing $0 in the Total field) are free. Please retain this printable license for your reference. No payment is required.** | |  |  |
|  | |  |  |
